# Computational Grounded Cognition: a new alliance between grounded cognition and computational modeling

**DOI:** 10.3389/fpsyg.2012.00612

**Published:** 2013-01-22

**Authors:** Giovanni Pezzulo, Lawrence W. Barsalou, Angelo Cangelosi, Martin H. Fischer, Ken McRae, Michael J. Spivey

**Affiliations:** ^1^Institute of Computational Linguistic “A. Zampolli,” National Research CouncilPisa, Italy; ^2^Institute of Cognitive Sciences and Technologies, National Research CouncilRome, Italy; ^3^Department of Psychology, Emory UniversityAtlanta, GA, USA; ^4^Centre for Robotics and Neural Systems, University of PlymouthPlymouth, UK; ^5^Division of Cognitive Sciences, University of PotsdamPotsdam, Germany; ^6^Department of Psychology, Social Science Centre, University of Western OntarioLondon, ON, Canada; ^7^Cognitive and Information Sciences, University of CaliforniaMerced, CA, USA

**Keywords:** grounding, embodiment, situatedness, cognitive robotics, situated simulation

## Abstract

Grounded theories assume that there is no central module for cognition. According to this view, all cognitive phenomena, including those considered the province of amodal cognition such as reasoning, numeric, and language processing, are ultimately grounded in (and emerge from) a variety of bodily, affective, perceptual, and motor processes. The development and expression of cognition is constrained by the embodiment of cognitive agents and various contextual factors (physical and social) in which they are immersed. The grounded framework has received numerous empirical confirmations. Still, there are very few explicit computational models that implement grounding in sensory, motor and affective processes as intrinsic to cognition, and demonstrate that grounded theories can mechanistically implement higher cognitive abilities. We propose a new alliance between grounded cognition and computational modeling toward a novel multidisciplinary enterprise: *Computational Grounded Cognition*. We clarify the defining features of this novel approach and emphasize the importance of using the methodology of Cognitive Robotics, which permits simultaneous consideration of multiple aspects of grounding, embodiment, and situatedness, showing how they constrain the development and expression of cognition.

## Introduction

Grounded theories increasingly challenge traditional views of cognition by proposing that the conceptual representations underlying knowledge are grounded in sensory and motor systems, rather than being represented and processed abstractly in amodal conceptual data structures.

The grounded perspective offers a unifying view of cognition. It stresses dynamic brain-body-environment interactions and perception-action links as the common bases of simple behaviors as well as complex cognitive and social skills, without ontological (or representational) separations between these domains (Spivey, 2007; Barsalou, 2008; Glenberg, 2010). Historically the grounded perspective has mainly targeted psychological phenomena. However, it aligns well with current theories in neuroscience that emphasize a continuity between the neuronal circuits that solve essential problems of action specification and selection in early organisms, and those that solve more elaborated problems in humans (cf. Shadlen et al., 2008; Cisek and Kalaska, 2010). Furthermore, the debate on the embodied nature of brain and cognition has a substantial impact on many other disciplines, including philosophy, linguistics, the social sciences, and robotics (Verschure et al., 2003; Pfeifer and Bongard, 2006; Clark, 2008).

Despite its growing popularity, the full potential of this methodology has not yet been demonstrated; and this is not only a matter of obtaining new empirical demonstrations of the importance of grounding for cognition. The framework is empirically well-established, but the theories are relatively underspecified. A real breakthrough might result from the realization of explicit computational models that implement grounding in sensory, motor and affective processes as intrinsic to cognition. In this article, we propose a new alliance between **grounded cognition** theories and computational modeling designed to work toward the realization of a novel multidisciplinary enterprise: ***Computational Grounded Cognition***.

## What is grounded cognition about? a field map

Grounded and embodied theories of cognition have become popular by stressing “the role of the body in cognition” (e.g., body orientation in relation to spatial processing or metaphorical reasoning, cf. Lakoff and Johnson, 1999). However, the scope of these theories is presently much wider and thus the literal meaning of the term “embodied cognition” is too narrow.

**Figure 1:**
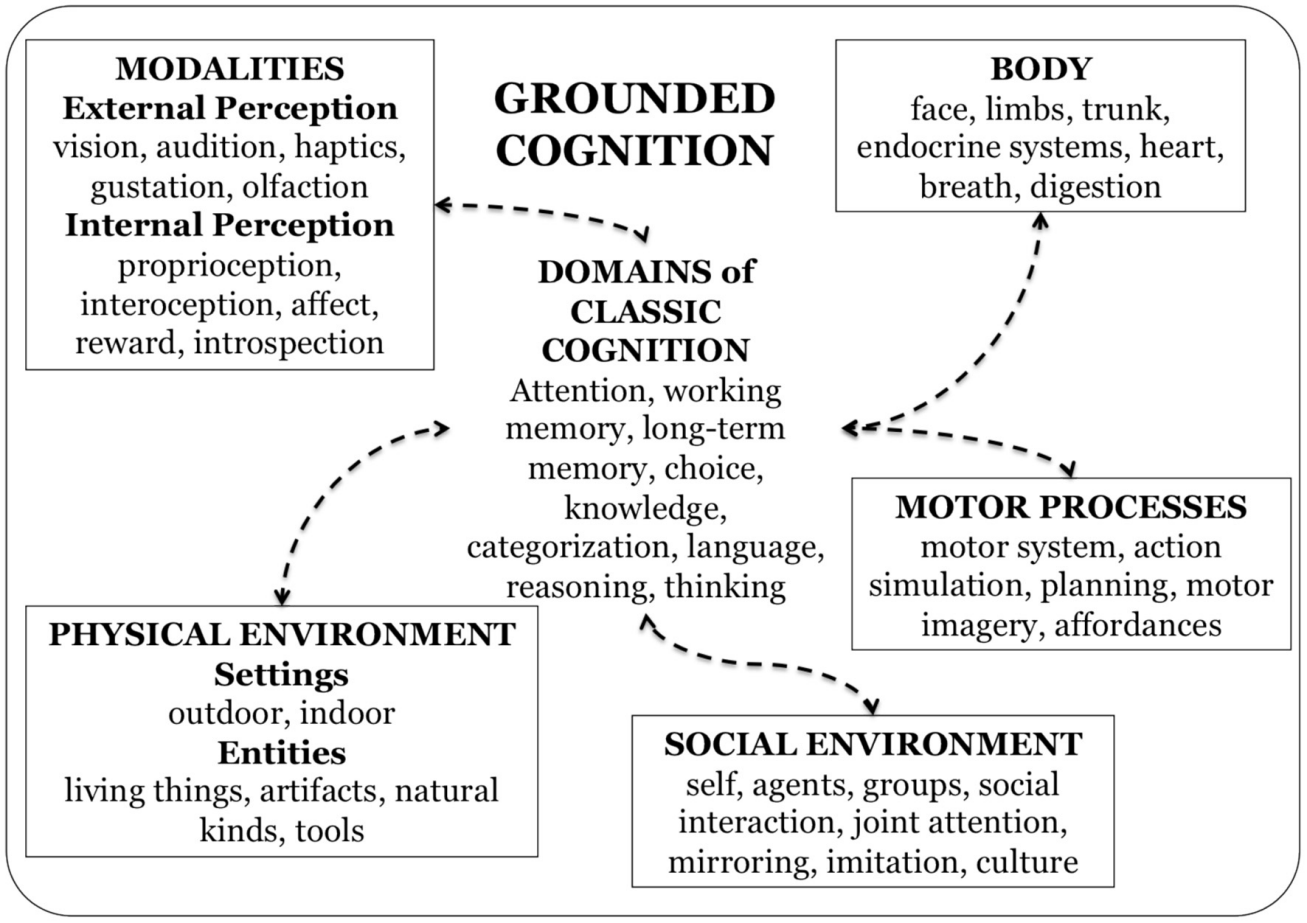
**Grounded cognition: a field map**.

As shown in Figure 1, *grounded cognition* is the name of a methodological approach to the study of cognition, which describes it as “grounded in multiple ways, including simulations, situated action, and, on occasion, bodily states” (Barsalou, 2008, pp. 619). Grounded cognition theories are bases for studying how knowledge and concepts are grounded in the modalities and bodily states, how cognitive processes such as language and thought are rooted in the body's interactions with the physical and social environment, and how *situated simulations* and the re-enactment of perceptual, motor and affective processes can support abstract thought.

Ultimately, the promise of grounded theories of cognition is to explain behavioral and experimental patterns in all of the traditional provinces of cognition (e.g., attention, memory, reasoning, and language) without recourse to a central module, showing instead that these patterns emerge from all of the systems in the brain, the body, and the environment (see the arrows pointing to the “Classic Cognition” domain in Figure 1). Below we provide five examples of grounded phenomena chosen for illustrative purposes.

**Figure 2:**
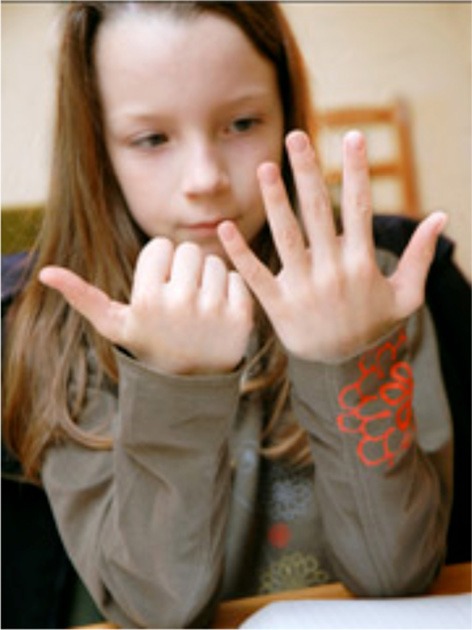
**Numerical cognition can be grounded in bodily actions**.

1. Barsalou and collaborators (Niedenthal et al., 2005; Wilson-Mendenhall et al., 2011) have documented that the acquisition of conceptual knowledge about emotions requires the embodiment of the corresponding bodily, affective, and emotional states. The same emotional states are re-enacted when emotion knowledge is used to perceive, recognize, and interpret the emotions of self and others. Niedenthal et al. (2005) discuss several other examples of social embodiment of attitudes, social perception, and emotion. (see also Ferri et al., 2010).
2. A significant example of embodiment signatures in cognition is attention deployment, which plays a central role in forming concepts and directing reasoning within a grounded cognition framework. For example, Grant and Spivey (2003) found that people who were about to solve a difficult diagram-based insight problem (Duncker's Tumor-and-Lasers problem) tended to produce subtly different eye-movement patterns compared to people who were about to give up on the problem. Those particular eye movements were part and parcel of the cognitive insight process itself, revealing that these people were about to discover the solution. In a second experiment, Grant and Spivey implicitly induced those eye movements in a new group of participants, and the number of people achieving insight doubled. Thus, the high-speed perception-action loop produced by eye movements (where an eye movement to an object influences both cognition and the next eye movement to another object, and so on) constitutes a sensorimotor ordering of micro-cognitive states from which high-level reasoning can emerge, such as abstract insight into a difficult puzzle.
3. Embodiment exerts its influence during development. Yu and Smith (2012) report that young learners solve the hard problem of learning object names by using an embodied strategy. Essentially, their hand, head and eye movements help to stabilize attention on a selected target, thus reducing competition in the visual field and ultimately supporting word learning.
4. The importance of the linkage between visual and motor processes is well-demonstrated by the stimulus-response compatibility effect (Tucker and Ellis, 2001; Ellis et al., 2007). These studies demonstrate that when we perform visual categorization tasks (e.g., identifying artifact vs. natural objects), the micro-affordances linked to the objects (e.g., power grasp for a large apple or precision grip for a small cherry) affect visual categorization performance, even if they are irrelevant for the task. This suggests that seeing an object automatically evokes motor programs appropriate to deal with it. Glenberg and Kaschak (2002) have provided additional demonstrations of *action-compatibility effects* (ACE), such as faster response times when the action used for reporting a choice is congruent with the context in which the action is typically used, or with the semantics and pragmatics of linguistic stimuli. This latter evidence suggests that even linguistic processing might be grounded in action (see also Glenberg, 2010).
5. Finger counting (see Figure 2) is used throughout the world to acquire conceptual knowledge about numbers. Historically, several cultures chose number symbols that resembled hand and finger shapes. Recently, the influence of finger counting habits on adult number processing was documented by Fischer (2008) and Domahs et al. (2010).

Note that we do not consider these (and other) grounding phenomena as optional add-ons of an overall amodal cognition, but *constitutive* of it. For instance, we do not assume that affective states *modulate* an amodal cognitive categorization process, but that they actually *constitute* the categorization, and thus are necessary for it (Niedenthal et al., 2005). This is a key difference between the grounded view and alternative theories that assign embodied phenomena a limited role in “true” cognition (e.g., those that assume amodal processing plus some contextual modulation), or those that assume a one-directional leakage from central amodal processing to the sensorimotor peripheries (Mahon and Caramazza, 2008). Further elaborations and distinctions, such as that between on-line and off-line embodiment, are discussed in Myachykov et al. (2013, unpublished).

### Grounding, embodiment, and situatedness: a conceptual clarification

**Figure 3:**
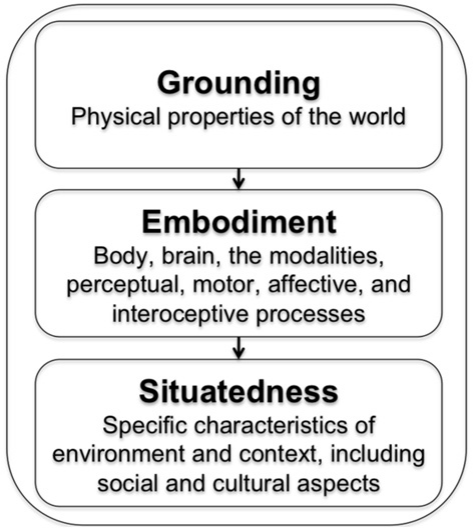
**Grounding, embodiment and situatedness: a cascade of effects on cognition**.

Grounded theories of cognition are often defined in contrast to traditional cognitive science (e.g., denying representation in terms of amodal symbols, and rejecting accounts of cognitive processing in terms of arbitrary symbolic manipulations), or reduced to a slogan (e.g., “the body plays a role in cognition”). The recent proliferation of grounded theories, which are often associated with different claims and perspectives (Wilson, 2002), has lead to a confusing usage of terms such as “grounded,” “embodied,” and “situated.” Like in Barsalou (2008), we use the term “grounding” as a suitcase word to define the field. Furthermore, as cognition can be grounded in multiple ways, it is often necessary to formulate more specific hypotheses, such as clarifying whether a cognitive process is influenced by a bodily state, a situated simulation, or both. For this, we propose that the effects of grounding, embodiment, and situatedness can be conceptualized as a cascade and have additive effects on cognition and representation (see Figure 3).

Below we give some examples of grounding, embodiment, and situatedness in the domain of numerical cognition. This domain is chosen because it has been considered as a domain par excellence for abstract and amodal concepts, a view we wish to challenge.

#### Grounding

Cognition has a physical foundation and is shaped by physical properties of the world, such as gravity, celestial light sources, and the laws of physics. Grounding of numerical cognition is reflected in the universal association of smaller numbers with lower space and larger numbers with upper space (Ito and Hatta, 2004; Schwarz and Keus, 2004), which reflects the physical necessity that aggregating more objects makes larger piles. Alternatively, grounding could be expressed through the perception of the cardinality of a set (Stoianov and Zorzi, 2012 and references therein) which natively supports fundamental cognitive operations.

#### Embodiment

Embodied representations are shaped by physical constraints of an individual's body. These sensory-motor experiences are structured according to physical principles that provide the grounding of cognition. Therefore, unusual bodies create unusual minds (Casasanto, 2011; Keehner and Fischer, 2012), and systematic use of one's body also influences the cognitive representation of numbers. Consider the fact that small/large numbers are responded to faster with the left/right hand. This SNARC effect (spatial-numerical association of response codes) is weaker in people who start counting on the fingers of their right hand (Fischer, 2008; Lindemann et al., 2011), presumably because right-starters have initially learned to associate small numbers with their right side. Importantly, it becomes clear that individual differences (physical and experiential) modulate this component of the SNARC effect.

#### Situatedness

Situated cognition refers to the context-dependence of cognitive processing, that is, to current constraints and task demands. Two examples illustrate how the specific situation further modulates the grounded and embodied representation of numbers (see also Fischer et al., 2009, 2010): first, a given number is associated with left or right space, depending on the number range tested (Dehaene et al., 1993). Second, turning one's head to the left/right induces the production of smaller/larger random numbers (Loetscher et al., 2008).

## Toward a computational grounded cognition

For grounded cognition theories to improve their explanatory scope, it is necessary to develop (better) *process models* of how grounded phenomena originate during development and learning and how they are expressed in on-line processing. Some examples of fruitful cross-fertilization between grounded cognition and computational modeling studies exist already (see e.g., Cangelosi and Riga, 2006; Spivey, 2007; Schöner, 2008; Hope et al., 2010; Pezzulo and Calvi, 2011), but the field would greatly benefit from an integrative effort: a *Computational Grounded Cognition* initiative.

Given that computational modeling has been around for a long time, what is special about Computational Grounded Cognition? Below we discuss this issue. First, we summarize the most important elements that *grounded computational models* should incorporate (see also Pezzulo et al., 2011). Second, we emphasize the importance of adopting **Cognitive Robotics** as a research methodology.

### Key assumptions of grounded computational models

Grounded computational modeling requires constructing models in which cognition is deeply interrelated with sensorimotor action and affect, and cognitive abilities emerge from the interactions between sub-processes rather than being implemented in isolated “cognitive modules.” Thus, the cognitive algorithms for word reading, speech recognition, object recognition, action understanding, and problem-solving should incorporate information from perceptual, motor, and affective processes when producing their results, and this is seldom seen in current computational models. This design method demands an integrative approach, which can be called “**interactionism**,” in which cognition stems from “coordinated non-cognition” (Barsalou et al., 2007).

#### Modal vs. amodal representations

According to grounded theories, cognition is supported by modal representations and associated mechanisms for their processing (e.g., situated simulations), rather than amodal representations, transductions, and abstract rule systems. Recent computational models of sensory processing can be used to study the grounding of internal representations in sensorimotor modalities; for example, *generative models* show that useful representations can self-organize through unsupervised learning (Hinton, 2007). However, modalities are usually not isolated but form integrated and multimodal assemblies, plausibly in association areas or “convergence zones” (Damasio, 1989; Simmons and Barsalou, 2003). Furthermore, during learning strong interdependences among sensory and motor representations are established that incorporate sensory regularities created by an agent's actions, forming sensorimotor contingencies (O'Regan and Noe, 2001) or emulators (Grush, 2004). Similarly, theories of active perception emphasize that sensory stimuli are not experienced passively but gathered actively, and action deployment structures the way people develop sensory representations. For example, Held and Hein (1963) showed that if cats experience the world only passively, they develop suboptimal perceptual representations. An open research objective is incorporating these ideas in computational models that realize the simultaneous grounding of representations in multiple modalities as well as across sensory, motor and affective processes.

One computational method for integrating modalities is designing robot controllers composed of multiple, interlinked modal maps (e.g., Kohonen's, 2001 self-organizing maps, or population codes, Ma et al., 2006; Morse et al., 2010). These might include motor maps, visual maps, and auditory maps, with the goal of investigating how they combine to support cognitive processing. This approach has recently been taken by Rucinski et al. (2011) when implementing the SNARC effect in a humanoid robot. Generative models can be used as well to explain multimodality and top-down influences (e.g., from motor or reward to sensory representations).

Another open research question concerns the hierarchical structure of association areas and the interaction of bottom-up activation vs. top-down simulation processes (Friston, 2010), which potentially permits conceptual knowledge to exert influence on sensorimotor processes.

#### From sensorimotor experience to cognitive skills: abstraction and abstract thought on the top of a modal system

Grounded computational models should not come pre-equipped with (arbitrary) representations. Instead, they should acquire “grounded modal symbols” through development and sensorimotor interaction, with genetic constraints presumably also playing a role. Furthermore, grounded computational models should acquire advanced cognitive abilities and abstract thought on top of their modal systems, not in separate subsystems. In other words, not only should representations be grounded in the modalities, but also their processing should be fully embodied, such that there is no central processing independent of sensorimotor processes and/or affective experience.

**Figure 4:**
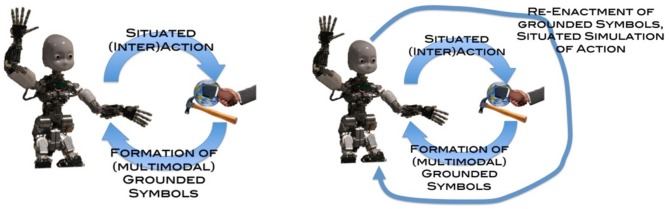
**A grounded cognition perspective on how grounded (modal) symbols are firstly formed based on situated interactions with the external environment, and later re-enacted as situated simulations, which afford higher-level cognitive processing**.

Figure 4 sketches an initial proposal for a “multi-modal processor” that can potentially implement cognitive and symbolic operations in modal systems (rather than with amodal symbols). First, grounded models are based on situated interaction of the robot with its environment, which can include both robots and humans. These multimodal symbols integrate perceptual, motor and valence information. Second, cognitive processing principally involves situated simulation, which is the re-enactment of grounded symbols (Barsalou, 1999). Cognition thus involves constraints that are similar to those in overt action because relevant episodic representations and associated bodily resources and sensorimotor strategies are activated.

Situated simulations may also support cognitive processing of objects or events in their absence. This can range from deliberate forms of imagery (Kosslyn, 1980) to automatic, unconscious processing (Jeannerod, 2001, 2006). Forms of action simulation and mental imagery have been linked to comprehension, reasoning, prospection, object categorization, action recognition, and other complex cognitive tasks (Glenberg, 1997; Jeannerod, 2006; Hassabis and Maguire, 2009; Moulton and Kosslyn, 2009; Schacter et al., 2012). These studies support simulation as a core process, which can both recreate experiences and productively recombine them, resulting in novel and prospective experiences, ultimately supporting off-line cognition without amodal representations.

An important challenge is explaining how abstract concepts and symbolic capabilities can be constructed from grounded categorical representations, situated simulations and embodied processes. It has been suggested that abstract concepts could be based principally on interoceptive, meta-cognitive and affective states (Barsalou, 2008) and that selective attention and categorical memory integration are essential for creating a symbolic system (Barsalou, 2003). Grounded computational models can help better specifying and testing these initial proposals.

#### Realistic linkage of cognitive processes with the body, the environment, and brain dynamics

In grounded cognition theories, cognitive processing in even abstract domains depends on sensorimotor skills and bodily resources. This leads to the possibility that expressions like “taking a perspective on a problem” or “putting oneself into another's shoes” or “grasping a concept” should be taken more literally than they normally are. This is due to at least two converging factors. First, cognitive abilities develop on top of the architecture for sensorimotor control, and the gradual maturation of the latter constitutes a scaffold for the former. Second, cognitive abilities re-enact and re-use modal representations rather than amodal recoding, and typically reuse existing sensorimotor competences in increasingly more complex cognitive domains (e.g., visuomotor strategies for problem solving, Grant and Spivey, 2003) rather than building novel modularized components. For these reasons, grounded cognitive processes have the same power, but also the same constraints, as bodily actions, and to understand the former it is necessary to provide realistic models of the latter.

Indeed, the grounded approach to modeling a cognitive ability begins by considering the sensorimotor processes that could support it. For example, a robot could first learn to spatially navigate, and then learn to reason in temporal and mathematical domains using its spatial representations and bodily processes as a scaffold. This potentially may produce behavior in line with the SNARC effect (cf. Rucinski et al., 2011). This approach enables incorporating increasingly more complex constraints as the tasks become more demanding. For example, spatial abilities can be reused to learn social interaction and joint action tasks. This approach can reveal how constraints from grounding, embodiment, and situatedness shape cognition in the brain. For example, studying language learning and usage in the context of realistic social interactions can help to explain why brain regions that are active when processing objects and actions are also active when nouns and verbs are comprehended (Pulvermüller, 2005).

Note that although we have mainly emphasized psychological processes, computational models benefit also from the incorporation of anatomical and neurophysiological constraints that help linking grounded theories to neuronal substrates. One example is the importance of reentrant loops and efference copies in neural computational hierarchies (Crapse and Sommer, 2008), which suggests a possible way that action streams influence perception and cognition. Recent EEG evidence has also shown that early sensory pathways are modulated by the action associations of objects and the intentions of the viewer (Goslin et al., 2012).

### Cognitive robotics as a research methodology for computational grounded cognition

Potentially, many types of computational models (e.g., connectionist, dynamical systems, and Bayesian) and “approaches” (see the recent dispute between “normative” and “emergentist” approaches, Griffiths et al., 2010; McClelland et al., 2010) are well-suited for modeling grounded phenomena. Shifting from a theoretician's to an engineer's perspective, one argument is that the most compelling demonstration of any theory's success is: when you build it, does it work?

A move from purely computational toward *cognitive robotic* models could drastically improve our ability to develop and test grounded theories of cognition. Cognitive robotics is a broad research area whose central aim is realizing complete robotic architectures that, on the one hand, include principles and constraints derived from animal and human cognition and, on the other hand, have realistic embodiment, sensors and effectors, and learn to act autonomously in complex, open-ended (and social). Cognitive robotics enables simultaneous consideration of multiple aspects of grounding, embodiment, and situatedness, showing how they constrain the development and expression of cognition. Thus, the benefits of adopting a cognitive robotics methodology are multifaceted.

1. Cognitive robotic models are an ideal choice to incorporate the entire cascade of effects of grounding, embodiment, and situatedness (including individual and social scenarios).
2. Cognitive robotics is suitable for experiencing environments full of choices, with numerous sources of rewards and punishments (e.g., manipulation of objects, social interactions). In turn, this permits linking behavior and cognitive processing with realistic motivational and emotional dynamics.
3. Cognitive robotic models favor unified design approaches that combine multiple psychological processes (e.g., attention, memory, action control) in the context of a specific task. This stands in opposition to (and usefully complements) the divide-and-conquer methodology of most empirical research, which, although useful, runs the risk of compartmentalizing cognitive phenomena. Cognitive robotics thus can be used to move beyond isolated models of single functions to focus on complete architectures that develop their skills over time.
4. Cognitive robotics incorporates nicely the idea that behavior and cognition are organized around the achievement of goals and the deployment of actions. The centrality of action and its linkage with perception emerge if one considers that for a functioning robot, action is also a way to change the environment in order to steer ensuing stimuli and to learn actively. Researchers from many traditions have emphasized the importance of learning from (the consequences of) one's actions. The goal-centered perspective could help with linking all cognitive abilities under common computational and neural processing principles. In this perspective, representation ability, memory ability, categorization ability, and attention ability, all could be ultimately in the service of action and goal achievement, rather than having disconnected functions (e.g., vision as a re-coding of the external world).
5. Cognitive robotic models can have realistic embodiment and can be used to investigate the reciprocal influences among the body, action and perception, such as, for instance, how action sculpts the body space (Rizzolatti et al., 1997). Principled approaches to perceptual processing describe the task of the brain as that of extracting statistical regularities from the sensorium. Cognitive robotics recognizes that also embodiment and action are sources of statistical regularities that are incorporated through learning (Verschure et al., 2003). Furthermore, embodiment can alleviate brain processing in multiple ways, for example by providing solutions to control problems (Pfeifer and Bongard, 2006), constraining choice to a space of possibilities that is body-scaled (Gibson, 1979), and implementing situated problem-solving strategies (Kirsh and Maglio, 1994). Note that to study most of these phenomena the mere possession of a body is not sufficient, but is necessary to develop realistic models of sensory processing, action systems, body schema and awareness (Haggard et al., 2002).
6. Cognitive robotic models are plausible platforms to explore the dynamics of change and adaptation at the evolutionary and developmental levels (studied by “developmental” or “epigenetic robotics”). At the evolutionary level, this can help us to understand how advanced cognitive skills develop on top of an existing neural architecture that solves basic problems of survival and reproduction (Pezzulo and Castelfranchi, 2007, 2009). At the developmental level, this can help us to understand how the progressive maturation at the level of sensorimotor coordination scaffolds the acquisition of cognitive and intellectual skills, and how the latter remain linked to the sensorimotor processes that provided scaffolding for their development (Rosenbaum et al., 2001; Thelen and Smith, 1994; von Hofsten, 2004); see also (Anderson, 2010) for a discussion of “reuse” of neural substrates across simpler and more complex abilities. **Developmental robotics** (specifically aimed at modeling developmental phenomena, see Cangelosi and Schlesinger, 2013) offers new ways to investigate this topic in more detail. For example, it permits manipulating robot knowledge and skills so as to assess what are the necessary prerequisites for the development of a particular cognitive ability. Furthermore, it permits studying the environmental conditions that facilitate or prevent normal cognitive development, and the social dynamics that scaffold language use and culture formation (Steels, 2003).

## Conclusions: challenges for a new scientific enterprise

Grounded theories of cognition claim that the development and expression of cognition is grounded in sensorimotor processes, affective states, and bodily strategies; furthermore, cognitive abilities have tight links with the environmental and social contexts in which they were acquired, rather than constituting isolated modules. A large body of work (reviewed in Barsalou, 2008) strongly supports this view. Still, grounded cognition theories lack process models and computational realizations.

We have proposed a “new alliance” of grounded cognition and computational modeling toward a novel scientific enterprise: *Computational Grounded Cognition*. This joint initiative requires that both computational modelers and empiricist adapt their research methodologies.

Computational modelers should undergo a “grounded turn”: they should fully incorporate the key tenets of grounded theories and increasingly adopt robotic platforms to better deal with issues of embodiment, situatedness, and development. Modeling grounded cognition tasks poses a huge multidimensional problem, as they could potentially incorporate multiple kinds of constraints (psychological, anatomical, physiological) and link to realistic physical and social environments (e.g., with human-robot interactions or multi-agent teams). This requires the elaboration of novel design methodologies that are *multilevel* and isolate sub-problems without losing the relevant directions. One possible starting point consists of designing functional-level architectures first, which more easily integrate processing principles, and then developing models that are more detailed at the neuronal level (see Verschure and Althaus, 2003). But clearly, the advantages of this approach (or the opposite one which starts from neurophysiologically detailed models) have to be evaluated in practice. One challenge for Computational Grounded Cognition is the realization of design principles that take multiple levels and constraints into account and allows researchers to study many phenomena, but are also specific enough to avoid losing contact with data obtained from animal or human experiments.

Empiricists should better incorporate the “synthetic methodology” of computational modeling and cognitive robotics within their own research. To this aim, it is necessary that good (off-the-shelf) *process models* of grounded phenomena become available that experimenters can easily incorporate in the design, operationalization and testing of their theories and experiments. Success stories already exist in many fields of psychology and neuroscience. For example, a family of reinforcement learning algorithms (e.g., Temporal Difference Reinforcement Learning, Sutton, 1988) and statistical methods (e.g., drift diffusion models, Ratcliff, 1978) are widely used to analyze neural data and to develop process models of (perceptual and reward-based) learning and decision-making. Similarly, the MOSAIC model provided a coherent framework for numerous experiments in computational motor control and social interaction (Wolpert et al., 2003). Similar proposals are emerging in the field of grounded cognition, especially in the dynamic systems family of models. From this perspective, cognitive processing does not operate by using symbols, but rather by the dynamic interactions of multiple processes of perception and action, which become coupled through learning and interaction with the environment. Spivey's (2007) proposal of continuous attractor dynamics constitutes a common computational foundation for numerous studies in attention, language processing, and reasoning, showing a continuous interaction between decision-making and motor execution. Computational models for the parallel specification and selection of multiple actions have been proposed in neuroscience that can explain the mechanics of decision-making and bridge the gap between simple and abstract choices (Cisek, 2012; Shadlen et al., 2008). Schöner's (2008) dynamic field framework is another example of a widely adopted approach that has the potential to explain cognitive phenomena at many levels. Important insights might come from recent advancements in probabilistic approaches to neural processing, biological learning, and control, as well (Doya et al., 2007; Friston, 2010; Shadmehr and Mussa-Ivaldi, 2012). Still, there is clearly a place for new ideas and ambitious researchers who want to develop better process models of cognition that have grounding, embodiment, and situatedness at their core.

### Conflict of interest statement

The authors declare that the research was conducted in the absence of any commercial or financial relationships that could be construed as a potential conflict of interest.

## Key Concept

Grounded Cognition: Grounded theories of cognition assume that all cognitive phenomena, including those traditionally considered the province of amodal cognition such as reasoning, numeric, and language processing, are ultimately grounded in (and emerge from) a variety of bodily, affective, perceptual and motor processes.
Computational Grounded Cognition: The fusion and cross-fertilization between grounded cognition theories, computational modeling, and cognitive robotics methods. The goal of Computational Grounded Cognition is developing process models of how grounded phenomena originate during (human and animal) development and learning and how they are expressed in on-line processing.
Cognitive Robotics: The field of robotics that takes inspiration from theories of human and animal cognition to endow robots with comparable cognitive abilities. Cognitive robotics ultimately aims at realizing intelligent and embodied systems (robots) that make decisions and take actions autonomously in realistic environments, including social (human-robot and robot-robot) scenarios.
Interactionism: A theoretical framework in the cognitive and neural sciences that places emphasis on how a given cognitive process (e.g., language, memory, numerical processing) emerges from the interaction between multiple subsystems rather than from the operation of an individual module devoted to that process.
Developmental Robotics: The field of robotics that takes inspiration from theories of human development to study the development of comparable cognitive abilities in robots. Developmental robotics ultimately aims at realizing robots that develop novel abilities by learning from exploration as well as with the help of teachers, much like children do.
